# Hearing Rehabilitation in Vestibular Schwannoma

**DOI:** 10.3390/audiolres13030031

**Published:** 2023-05-12

**Authors:** Gauri Mankekar, Sean Holmes

**Affiliations:** 1 Department of Otolaryngology, Louisiana State Health University Sciences Center, Shreveport, LA 71103, USA; 2 Department of Otolaryngology, Cox Health Medical Group, Springfield, MO 35807, USA

**Keywords:** vestibular schwannoma, CROS, BiCROS, BAHS, ABI, cochlear implant

## Abstract

The most common complaint among patients with vestibular schwannoma (VS) is hearing loss. This significantly affects the quality of life before, during, and after treatment for patients with VS. Untreated hearing loss in VS patients may even lead to depression and feelings of social isolation. A variety of devices are available for hearing rehabilitation for patients with vestibular schwannoma. These include contralateral routing of hearing signals (CROSs), bone-anchored hearing devices, auditory brainstem implants (ABI), and cochlear implants. In the United States, ABI is approved for patients 12 years of age and older with neurofibromatosis type 2. In the past few years, cochlear implantation has been offered simultaneously or sequentially with tumor resection or irradiation, or even to patients whose VS have been monitored with serial imaging. However, determining the functional integrity of the auditory nerve in patients with vestibular schwannoma is a challenge. This review article consists of (1) the pathophysiology of vestibular schwannoma (VS), (2) hearing loss in VS, (3) treatment of VS and associated hearing loss, (4) options for auditory rehabilitation in patients with VS with their individual benefits and limitations, and (5) challenges in hearing rehabilitation in this cohort of patients to determine auditory nerve functionality. (6) Future directions.

## 1. Introduction

**Figure 1:**
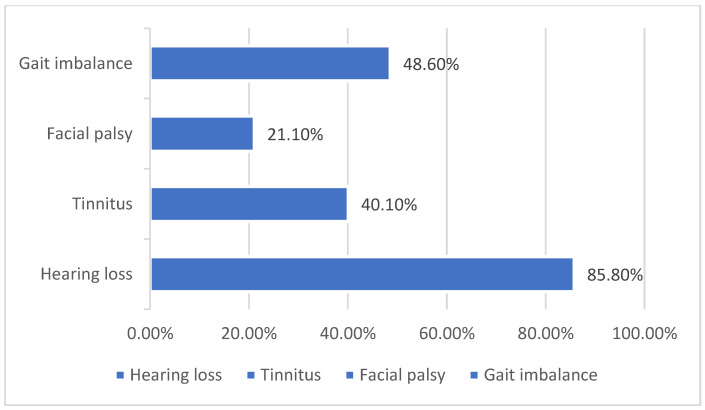
Common clinical presentations in patients with VS [3].

Vestibular schwannomas (VS) are benign neoplasms that arise from Schwann cells surrounding the superior or inferior vestibular (eighth cranial) nerve [1,2]. Tumors can be present in a number of intracranial or extracranial locations, thus making clinical symptoms at the time of presentation vary significantly. Symptoms at presentation may include any combination of the following: sudden sensorineural hearing loss, progressive hearing loss, central vestibular weakness, peripheral vestibular weakness, tinnitus, facial numbness, or facial weakness. In a study of more than 1000 patients, 85.8% had hearing loss, 48.9% had paresthesia of the face, there was a gait imbalance in 48.6%, and 40.1% had tinnitus [3] (Figure 1). The Characterization of these functional deficits at the time of initial work is imperative, not only in treatment planning but also in the determination of the most effective rehabilitative options as well [4]. There are not only many different treatment options for VS, but also many different options for subsequent or simultaneous auditory rehabilitation as well. New technologies are being developed and improved upon daily to better assist VS patients before, during, and after their treatment. The functionality of many of these implantable devices depends on the integrity of the cochlear nerve during and after treatment, which has remained a challenge to assure as a result of the nature of this disease process.

VS can involve a number of different anatomic locations at presentation, including the cerebellopontine angle (CPA), intracanalicular (IC), or intralabyrinthine (IL). While early tumors are frequently isolated to one of these locations, more advanced tumors commonly extend to involve multiple locations. Magnetic resonance imaging (MRI) with and without gadolinium is typically used to detect and characterize these tumors, which dictate treatment and rehabilitative options. The incidence of VS appears to have increased, which may be explained by the increased accessibility to MRI and screening of patients with asymmetrical and/or sudden sensorineural hearing loss [1,2]. Less than a century ago, tumors such as this were known to cause complete deafness and vestibular weakness despite treatment in most cases, and furthermore, the options available for auditory rehabilitation were minimal at best. With the increasing incidence of this disease process and the drastically improving technologies that widen options for auditory rehabilitation, the future of treatment and quality of life for our VS patients is more exciting than ever.

Approximately 10% of all intracranial lesions are comprised of VS [4] and 95% of VSs are sporadic and occur unilaterally. Bilateral VS occurs in approximately 5% of cases, which represents the NF2 population [5,6]. The most common overall initial presenting symptom that patients complain of when they have VS is hearing loss, which is seen in more than 90% of cases. The type of hearing loss is typically sensorineural and occurs on the side of the sporadic VS [7]. Approximately 12–26% of patients with VS develop sudden sensorineural hearing loss at some point during the course of their disease [8,9]. In addition, a recent study reported a long-term risk of progression of hearing loss in the contralateral ear in patients with unilateral VS [10]. In patients with neurofibromatosis type 2 (NF-2), 90% of the patients develop bilateral vestibular schwannomas, leading to bilateral hearing loss at a young age [11,12]. In addition to bilateral cochlear and/or retrocochlear hearing loss, NF2 patients with VS typically also can have meningiomas, other intracranial tumors, hydrocephalus, and posterior subcapsular lenticular opacities [13]. 

Treatment of sporadic VS consists of a wait-and-scan observation approach or surgical resection or irradiation. The treatment modality depends on the patient’s choice, tumor characteristics (e.g., size of the tumor, age of the patient, rate of tumor growth, cystic or solid components of the tumor, presence of serviceable hearing at the time of diagnosis, and associated comorbidities). Irradiation of the tumor can be performed with either stereotactic radiosurgery (SRS), gamma knife surgery (GKS), or fractionated stereotactic radiotherapy (FSRT) [14]. The goal of treatment in small and medium-sized VS is hearing preservation [11,12]. There is a high risk of cochlear nerve injury and/or damage to the cochlea itself from the combined effects of the VS and current treatment modalities, including surgery and radiation. In sporadic VS as well as NF2 patients with VS, hearing loss as a result of the tumor or therapy can influence their quality of life significantly. After radiotherapy, hearing loss in VS patients has been reported to progress gradually, with only 23% of patients with VS having serviceable hearing at 10 years postradiotherapy [15]. Progressive decline in hearing is also demonstrated in patients with stable tumors being observed with serial magnetic resonance imaging, especially when hearing loss is present at the initial diagnosis [16,17]. 

Hearing rehabilitation is extremely important for all patients with VS to improve their quality of life. However, it is especially important for patients with NF2-associated VS, as they can develop progressive visual disturbances as well as hearing loss. The only available treatment option historically was an auditory brain stem implant (ABI). Today, several options for hearing rehabilitation, besides ABI, can be offered to VS patients that suffer from hearing loss due to the natural history of the tumor or as a result of the treatment modalities. These include conventional hearing aids, contralateral routing of signal (CROS) hearing aids, bone-anchored hearing systems (BAHSs), and cochlear implants (CIs). This review will discuss the benefits and limitations of each of these different auditory rehabilitation options as we seek to improve our ability to affect the quality of life for our VS patients during and after their treatment. 

## 2. Nonsurgical Options for Auditory Rehabilitation: Conventional Hearing Aids, Contralateral Routing of Signals (CROS), and Bilateral Contralateral Routing of Signal (BiCROS) Hearing Aids

Patients with unilateral vestibular schwannoma typically present with asymmetrical sensorineural hearing loss in the affected ear with normal hearing thresholds in the unaffected ear, poor speech discrimination, and tinnitus [18]. Conventional hearing aids are offered to patients with vestibular schwannoma if they have mild to moderately severe sensorineural hearing loss. However, conventional hearing aids offer little to no benefit to patients with severe to profound unilateral hearing loss. For these patients, CROS hearing aids are better options for hearing rehabilitation. 

CROS hearing aids are used to route sound from the worse-hearing ear to the better-hearing ear and are typical for patients with single-sided deafness. Two different internal devices within the hearing aids themselves are used to execute this contralateral routing process. The first device is inside the hearing aid, which is placed in the worse-hearing ear. It has a microphone that detects sound from that worse-hearing side; however, instead of routing that signal into the worse-hearing ear, similar to what a traditional hearing aid would do, the signal is routed to the opposite (the better-hearing) ear using a transmitter. Thus, sounds and information from the side of the worse-hearing ear are processed through the better-hearing ear.

BiCROS is recommended for patients with mild-to-moderate hearing loss in the contralateral ear, as compared to a normal-hearing contralateral ear for CROS. Historically, only a small percentage of patients with single-sided deafness (SSD) reported improvement in their quality of life with the use of CROS or BiCROS [19]. This may be due to the inherent limitations of sound/information transfer by air conduction as compared to bone conduction systems or electrical signal conduction systems, both of which have a greater capacity to send a stronger signal into the auditory centers for interpretation.

Advances in CROS hearing aid system technology have addressed previous limitations, such as short battery life, poor sound quality, discomfort, and aesthetics due to their large size. The addition of wireless streaming allows the sound signal to be transmitted to the better-hearing ear without delays or audible interference [13]. Advanced algorithms have allowed for improved noise reduction, and directional microphones with automatic adaptive directionality have improved the signal-to-noise ratio for users [14,15]. This has led to patients with VS accepting and increasing their usage of CROS and BiCROS devices as compared to previous adaptations of these devices. However, CROS and BiCROS do not provide binaural hearing and can even disrupt the individual’s use of monaural spectral cues [20,21]. Surveys of vestibular schwannoma patients have reported that 30–32% of patients used conventional hearing aids, 23–30% used CROS devices, and 21.7% used BAHS [17,22]. 

## 3. Bone Anchored Hearing System (BAHS)

Bone-anchored hearing systems are surgical solutions used to mitigate unilateral hearing loss in patients with VS. It consists of a titanium screw or plate fixed to the bone behind the ear, and it connects either percutaneously or transcutaneously to an external bone oscillator. The microphone, which receives the sound signal, is integrated into the bone oscillator. The sound processor transforms the signal into an oscillatory signal, which is transmitted through the plate/screw, which in turn causes the bone to vibrate at a certain frequency and send a signal into the cochlea on the better hearing side. Unlike the CROS hearing aid, which uses air conduction, the BAHS uses bone conduction for sound transmission. 

BAHS works best for patients with VS who have good contralateral hearing. Additionally, similar to CROS hearing aids, BAHS is limited by its inability to provide binaural hearing, which is important for sound localization and improving speech understanding in noise [19]. However, of note, due to the nature of bone conduction over air conduction, the high-power BAHS devices are able to provide power signal input through bone conduction. Surgery is overall safe and confers less risk than the implantation of electrical devices, such as CI or ABI, as no inner ear structures are opened during BAHS surgery. Additionally, there are surgical and nonsurgical treatment options for BAHS, which are interesting treatment options for all sorts of VS patients. 

The wide variety of BAHS devices may be categorized into percutaneous or transcutaneous, and within the transcutaneous category, there are surgical and nonsurgical options. The variety of BAHS devices that are available, make for many different rehabilitative options for both pediatric and adult VS patients, as well as those who are unable to undergo surgery.

## 4. Auditory Brain Stem Implant (ABI)

ABI is a surgically implanted device that bypasses the cochlear nerve to electrically stimulate the neurons in the brain stem dorsal cochlear nucleus. For a long time, ABI was the only treatment for hearing rehabilitation for patients with hearing loss caused by VS. The ABI electrodes are embedded in a silastic paddle and placed in the fourth ventricle. The device has an external battery, a microphone, a speech processor, a transmitter coil, and a magnet that is worn behind the ear. ABI has been approved by the US FDA for use in patients age 12 and older with NF2 for hearing rehabilitation. Patients with ABI typically have sound awareness, but results with ABI have been modest, and open-set speech (conversational speech in daily life) after ABI is an exception [23,24]. Less than 5% of NF2 patients have open-set speech. It can assist lip reading, enable the identification of environmental sounds, and can drastically improve the patient’s quality of life [25,26].

ABI is a great treatment option when the cochlear nerve has been rendered absent or nonfunctional; however, determining the status of the cochlear nerve remains a challenge. We must establish a standardized method of testing the cochlear nerve for functional integrity during and/or after observation or treatment for VS. This will allow a better treatment algorithm to be established and help us determine exactly who might benefit from CI over ABI. Since ABI surgery is more invasive than CI surgery, it should only be considered in appropriate patients who would not benefit from CI. Currently, though, our ability to make that determination on a consistent basis remains somewhat limited.

## 5. Cochlear Implant (CI)

Cueva et al. in 1992 reported data from six patients with grossly intact cochlear nerves and anacusis after resection of VS who were able to perceive sound following the electrical promontory stimulation. They postulated that these patients could potentially benefit from cochlear implantation [27]. Subsequently, Hoffman et al. reported a case of a young NF2 patient who lost his hearing despite hearing preservation with retrosigmoid resection of the VS with sparing of the cochlear nerve. The patient underwent cochlear implantation three months after VS resection and achieved open-set speech [28].

Since then, several studies have demonstrated CI to be an effective means for auditory rehabilitation in patients with sporadic unilateral VS as well as NF2 patients after microsurgical resection or radiation therapy [4,19,28,29,30,31,32]. There are also studies that demonstrate that CI is an effective option for hearing rehabilitation in patients with small/stable VS who are being followed with serial imaging [17,33,34].

The CI electrically stimulates the cochlear nerve in the inner ear to enable identification, interpretation, and associating meaning with the sound (auditory perception). This is unlike the ABI, which stimulates the neurons of the dorsal cochlear nucleus in the brain stem. CI is a device consisting of a surgically implanted electrode array with a receiver-stimulator coupled with an external sound processor. The microphone on the external processor receives sound and converts it to an electrical signal, which is carried to the spiral ganglion cells of the cochlear nerve via a multielectrode array. Cochlear implant electrode arrays utilize the tonotopic organization of the cochlea to maximize sound intelligibility by covering almost the entire spectrum of perceivable auditory frequencies. For a cochlear implant to be effective, cochlear nerve integrity is essential and may play a role in hearing outcomes after CI in VS patients. This means that any therapeutic modality offered to treat VS must also be able to preserve cochlear nerve integrity. Even if the cochlear nerve is preserved during surgical resection, it has been shown that a majority of those patients can go on to lose their hearing [23]. Several factors, such as vascular compromise, trauma to the nerve, and cochlear fibrosis, can influence conduction along the cochlear nerve after surgical resection and the ability of the patient to benefit from a cochlear implant [23].

Determining the functional integrity of the cochlear nerve has always presented a challenge. The electrical promontory stimulation (EPS) test is a test that has been used traditionally to determine the functionality of the cochlear nerve. It is performed by placing a transtympanic needle electrode on the surface of the cochlea, and the cochlea nerve is electrically stimulated through the monitoring of subjective and electrically evoked auditory brainstem responses. A loss of neural integrity at the spiral ganglion or cochlear nerve level is indicated by an absence of response to stimulation. On the other hand, the presence of a response is indicative of vascular compromise of the cochlea with the sparing of the spiral ganglion and the cochlear nerve. This test has its drawbacks, as some patients with good EPS do not benefit from a CI, while some with poor EPS have achieved open-set speech with a CI [23,27,29].

In a large systematic review, 65% of patients with NF2 who underwent surgical resection with ipsilateral cochlear implantation were found to have developed open-set speech with the CI [32]. Interestingly, no differences in outcomes after CI were observed when comparing simultaneous to sequential tumor resection and implantation [32]. In another systematic review performed by Wick et al., CI performance was not found to be influenced by the timing of cochlear implantation, whether during or after tumor resection [23]. In an ongoing FDA-approved clinical trial, a device monitoring the auditory nerve during VS resection called the auditory nerve test system (ANTS) is helping the surgeon-investigators preserve the nerve during VS resection and rehabilitate patients with simultaneous cochlear implants [35]. 

## 6. Discussion

VS are benign, slow-growing tumors. Their growth pattern causes a mass effect on surrounding neural structures. Patients commonly have hearing loss. Sporadic VS is unilateral versus bilateral in patients with neurofibromatosis type 2. Hearing loss in these patients can occur due to compression of the auditory nerve by the tumor with subsequent cochlear dysfunction [36], molecules secreted by the schwannoma [37,38], or a schwannoma-associated inflammatory response [39]. The severity of hearing loss in patients with VS ranges from normal hearing to profound sensorineural hearing loss. Sudden sensorineural hearing loss has been reported in approximately 10–30% of VS patients [8,40]. It has been reported that 3–5% of patients with sudden hearing loss go on to receive a diagnosis of VS [41]. A recent study reported that the recovery rate of VS-associated sudden hearing loss decreases with increasing recurrent episodes of sudden loss [42].

Hearing loss and tinnitus in sporadic and NF2 patients affect their quality of life irrespective of whether the treatment approach is observational, surgical resection, or radiation. The wait-and-scan approach is recommended for VS less than 1.5 cm in diameter, with a slow or no growth pattern, or in elderly patients who do not want to undergo surgery. Studies have reported irreversible hearing loss in these patients, even during observation [43]. Ariano et al., in their review, mentioned that the growing objective of surgical resection of VS is hearing preservation, with retrosigmoid and middle fossa approaches becoming more popular as they are associated with hearing preservation [44]. Surgical resection is recommended for large tumors causing brain stem compression, especially in young patients. Stereotactic radiation is another modality of treatment for small to medium-sized VS, especially in older patients and in patients who do not have symptoms of brainstem compression. Better functional outcomes have been reported in appropriately selected patients with stereotactic radiosurgery [45]. Studies have reported a gradual decrease in hearing after radiosurgery [15,46], and loss of serviceable hearing in most patients over a period of ten years poststereotactic radiosurgery [47]. 

CROS, BiCROS, BAHS, CI, and ABI are all feasible options in patients with sporadic VS and NF2 when appropriately selected to address the severity, type of patient’s hearing loss, and integrity of the cochlear nerve after tumor resection (Table 1). Hearing rehabilitation can help improve the patient’s quality of life. However, there are several challenges to hearing rehabilitation, and these can be divided into patient-related factors, determining the functional integrity of the cochlear nerve, and bypassing the cochlear nerve with ABI.

In a survey of patients with unilateral VS, the majority (88%) of the patients reported being able to hear “poorly” or “not at all” in the ipsilateral ear. However, the authors found that less than one-third of the patients pursued hearing rehabilitation. They were neither bothered by their hearing loss nor aware of the options for hearing rehabilitation [17]. Approximately 62% of surveyed respondents in another study did not use hearing assistive devices at the time of the survey [22]. Many patients had no recollection of hearing rehabilitation counseling. Frequent and more involved counseling about hearing rehabilitation may be helpful in improving the quality of life of VS patients [17]. The prohibitive cost of hearing rehabilitation is another important patient-associated factor. Patients’ insurance often does not cover hearing amplification devices, and the patients are unable to afford the appropriate amplification devices out of pocket. Additionally, in patients with poor speech discrimination, hearing amplification may not provide adequate benefit, leading patients to avoid it altogether. 

For a long time, ABI was the only treatment option for hearing rehabilitation for VS patients with hearing loss. They are implanted simultaneously or sequentially after tumor resection. They can help VS patients lip read and identify environmental sounds, but less than 5% of NF2 patients have open-set speech [23,24]. In the last decade, simultaneous as well as sequential cochlear implantation after tumor resection has gained popularity. A systematic review by Wick et al. suggests the feasibility of cochlear implantation after VS resection, with 54.8% of patients achieving open-set speech [23] with cochlear implants, unlike CROS and bone-anchored hearing systems, which help to improve speech understanding in noise and restore binaural hearing, thus enabling sound localization [23]. 

The cochlear nerve must be anatomically and functionally intact for the cochlear implant to provide benefit to the patient. One of the main limitations of CI in the hearing rehabilitation of patients with VS continues to be the assurance of the functionality of the cochlear nerve. Several studies are ongoing to develop a testing method for monitoring auditory nerve function in these patients [32,45,47]. Medina et al. have suggested the use of an intracochlear test electrode (TE) to elicit evoked brainstem responses (EABR) after VS removal to determine the integrity of the auditory nerve [47].

A systematic review of various cochlear monitoring techniques, such as auditory brainstem responses (ABR), direct eight cranial nerve monitoring (DENM), distortion product otoacoustic emissions (DPOAE), and postauricular muscle responses (PAMRs), reported that the outcomes of cochlear nerve monitoring were satisfactory with all techniques. However, none of the techniques could be rated superior to the others due to the heterogeneity of reporting the outcomes of the techniques in the literature [45]. To determine the ideal technique to determine auditory nerve functionality and serviceable hearing, standardized studies will be needed.

## 7. Conclusions

The devices/options for auditory rehabilitation in VS patients vary greatly, however, they can be grouped into three categories: (1) air conduction devices, including CROS and BiCROS hearing aids, (2) bone conduction devices, for which there are surgical and nonsurgical modalities, and (3) electrical conduction devices, which include ABI and CI. It is important to have different treatment options to fit the needs of each individual patient. By increasing awareness of the options for auditory rehab in VS patients, we may improve our ability to assist this patient population through the exciting technologies that have been developed and employed in recent years.

Of all available options for auditory rehabilitation in VS patients, CI currently has the most potential for improvement in open-set speech, improvement to speech understanding in noise, and improvement to sound localization/binaural hearing. Currently, though, our ability to offer CI to our VS patients is mainly limited by concerns about the current or future status of their cochlear nerve, whether they undergo observation or treatment. Technologies have been developed to confirm cochlear nerve functionality intraoperatively, which has changed our approach to auditory rehabilitation in VS patients. As a result of this technology, patients have been able to receive simultaneous tumor resection and cochlear implantation, which has demonstrated promising and interesting results to date.

## 8. Future Directions

Multiple other neoplastic conditions elsewhere in the body are treated with simultaneous resection and rehabilitation. This type of treatment planning is unique in that it not only leads to tumor control, but simultaneously improves the quality of life for our patients as they go through these debilitating conditions, such as VS. Further research is needed to better standardize the testing for the functional integrity of the cochlear nerve after resection or radiation in hopes of improving hearing rehabilitation options for patients with both unilateral and bilateral VS in the future.

Something that will be imperative in helping maximize the number of patients that may benefit from simultaneous resection and CI implantation in the future is diagnosing these tumors in a timely manner and moving forward with treatment while hearing remains serviceable. As such, MRI imaging for at-risk patients remains critical to the care of this patient population.

As implantable/wearable device technology continues to improve, it continues to interface not only with the human body more effectively, but also with other forms of technology in our environment as well, and this will one day make the rehabilitative process for our VS patients more seamless than ever as they go through surveillance and/or treatment and recovery.

## Figures and Tables

**Table 1:** Advantages and disadvantages of each of the modalities for auditory rehabilitation during and/or after VS observation and/or treatment.

Hearing Rehabilitation Options	Figures	Advantages	Disadvantages
CROS/BiCROS	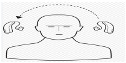	Nonsurgical option. Improved noise reduction	Does not provide binaural hearing
BAHS	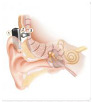	Nonsurgical and surgical options available	Does not provide binaural hearing
CI	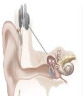	Open-set speech results in carefully selected VS and NF2 patients	Surgical options for cochlear nerve integrity and functionality are essential for success
ABI	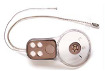	Option for patients who are not candidates for CI	Results are modest

## Data Availability

Not applicable.
